# Effect of Jiuwei Zhenxin granules on serum TREM1 expression and its regulatory role in depression and anxiety in patients with coronary heart disease

**DOI:** 10.1097/MD.0000000000044143

**Published:** 2025-09-05

**Authors:** Yi Xiang, Shan Hui, Zhongguang Yan, Hao Nie, Chun Guo

**Affiliations:** a Department of Cardiology, Hunan Provincial People’s Hospital (The First Hospital Affiliated with Hunan Normal University), Changsha, China; b Department of Geriatrics, Hunan Provincial People’s Hospital (The First Hospital Affiliated with Hunan Normal University), Changsha, China; c Pediatric Laboratory, Hunan Provincial People’s Hospital (The First Hospital Affiliated with Hunan Normal University) Laboratory Physician, Changsha, China.

**Keywords:** anxiety, coronary heart disease, depression, Jiuwei Zhenxin granules, prognosis, TREM1

## Abstract

This study explored the effects of Jiuwei Zhenxin (JWZX) granules on serum triggering receptor expressed on myeloid cells 1 (TREM1) levels and their role in regulating depression and anxiety in patients with coronary heart disease (CHD). A total of 100 CHD patients were enrolled from January 2020 to January 2023: 50 received JWZX granules and 50 underwent conventional treatment. Clinical data and psychological scores were collected. Serum TREM1, brain-derived neurotrophic factor, and 5-hydroxytryptamine (5-HT) levels were measured by enzyme-linked immunosorbent assay before and after 3 months of treatment. Major adverse cardiovascular events were tracked during 1-year follow-up. JWZX significantly reduced Hamilton Anxiety Scale, Hamilton Depression Scale, Self-rating Anxiety Scale, Self-rating Depression Scale, Generalized Anxiety Disorder Scale-7, and Patient Health Questionnaire-9 scores. Compared to controls, the JWZX group showed lower TREM1 and higher brain-derived neurotrophic factor and 5-HT levels. TREM1 was negatively correlated with 5-HT and positively with depression scores. Receiver operating characteristic analysis showed TREM1 had moderate predictive value for major adverse cardiovascular events (area under the curve: 0.689; cutoff: 66.39 pg/mL). Logistic regression identified TREM1, 5-HT, and JWZX as significant predictors of prognosis. JWZX granules improved mood symptoms and prognosis in CHD patients by modulating TREM1 and related biomarkers. Serum TREM1 may serve as a biomarker for cardiovascular risk and treatment response.

## 1. Introduction

Cardiovascular disease (CVD) is virtually the leading cause of death in every region around the world.^[1]^ According to statistics from the World Health Organization in 2015, CVD accounted for 17.7 million deaths, approximately 31% of the global mortality rate. Among them, an estimated 7.4 million deaths were attributed to coronary heart disease (CHD).^[2,3]^ Risk factors for CHD include age, gender, smoking status, psychosocial factors, diabetes, hypertension, body mass index, alcohol consumption, regular physical exercise, and daily intake of fruits and vegetables.^[4,5]^ Additionally, coronary artery syndrome, usually caused by coronary artery stenosis or obstruction leading to myocardial ischemia, is a major risk factor for CHD.^[6]^ In China, the prevalence and mortality rates of CHD continue to rise, imposing a significant economic and public health burden on society, necessitating further exploration of treatment approaches for patients.^[7]^

In recent years, research has found that patients with CHD often experience psychological health issues. For instance, a meta-analysis of 23 studies conducted in Chinese hospitals found a depression prevalence rate of 51% among hospitalized CHD patients.^[8]^ Another study discovered that the prevalence of depression among Chinese patients with acute myocardial infarction was 21.7%, significantly higher than the control group (10.4%).^[9]^ Evidence also suggests that approximately 31.3% of patients with depression present with cardiovascular disorders as the primary symptom, indicating a close relationship between CHD and mental health.^[10]^ New evidence confirms that anxiety and depression are significantly associated with an increased risk of subsequent cardiovascular events.^[11]^ These studies all indicate the importance of addressing the psychological health of CHD patients alongside their treatment.

Jiuwei Zhenxin (JWZX) granules, a traditional Chinese medicine composed of ingredients such as ginseng, sour jujube seed, schisandra, poria, polygala, incised notopterygium, dwarf lilyturf tuber, prepared rehmannia root, and cinnamon, are used for the treatment of generalized anxiety disorder with heart-spleen deficiency. The symptoms include excessive worrying, insomnia or vivid dreams, palpitations, poor appetite, mental and physical fatigue, dizziness, excessive sweating, frequent sighing, sallow complexion, pale tongue with thin white coating, and wiry or deep thready pulse.^[12]^ It has been reported that JWZX granules can alleviate anxiety symptoms in patients and promote neural function recovery. Its mechanism of action may involve regulating neurotransmitters to inhibit autonomic nervous system dysfunction, thus reducing anxiety.^[13]^ However, there is currently no clinical research focusing on the impact of JWZX granules on psychological factors in CHD patients.

Triggering receptor expressed on myeloid cells 1 (TREM1) is a pro-inflammatory receptor primarily expressed on monocytes and neutrophils. It plays a crucial role in amplifying inflammatory responses and has been implicated in various inflammatory and cardiovascular diseases.^[14]^ Recent studies have suggested that TREM1 is also involved in the pathogenesis of depression and anxiety. Elevated levels of TREM1 have been associated with increased neuroinflammation, which is a key factor in the development of depressive and anxiety disorders.^[15]^ Furthermore, TREM-1 inhibition has been shown to alleviate depressive-like behaviors by mitigating neuroinflammation in the prefrontal cortex through the PI3K/Akt signaling pathway, suggesting that TREM-1 may play a critical role in the pathophysiology of depression.^[16]^ However, there is currently no clinical research focusing on the expression of TREM1 in CHD patients and its potential role in the psychological health of these patients.

This prospective observational study aims to investigate the impact of JWZX granules on serum TREM1 expression and its regulatory role in depression and anxiety in patients with CHD. By exploring the relationship between TREM1 levels and psychological factors, we hope to provide new insights into the mechanisms underlying the psychological health issues of CHD patients and offer potential therapeutic targets for improving their prognosis.

## 2. Methods

### 2.1. Participants

This prospective observational cohort study enrolled 50 CHD patients treated with JWZX granules and 50 CHD patients received conventional treatment from January 2020 to January 2023. All patients underwent percutaneous coronary intervention (PCI) surgery under the same treatment team in our hospital. The inclusion criteria were as follows: in accordance with the “2013 ESC guidelines on the management of stable coronary artery disease” for selection criteria.^[17]^ Underwent PCI surgery in our hospital. Patients could independently complete psychological questionnaires including the Hamilton Anxiety Scale (HAMA), Hamilton Depression Scale (HAMD), Self-rating Anxiety Scale (SAS), Self-rating Depression Scale (SDS), Generalized Anxiety Disorder Scale-7 (GAD-7), and Patient Health Questionnaire (PHQ-9). Patients had at least 1 year of follow-up. The exclusion criteria were as follows: patients with severe liver or kidney diseases, traumatic brain injury, or malignant tumors. Patients with bipolar affective disorder, depression, or anxiety disorders before PCI. Patients taking antipsychotic, antidepressant, or antianxiety medications continuously. Patients with other severe mental illnesses, including schizophrenia or severe dementia. Patients who did not take JWZX granules as instructed in the product manual. The study was approved by the Ethics Committee of our hospital. All patients signed an informed consent.

### 2.2. Treatment

The patients in the conventional treatment group received standard treatment, including nitrate esters, beta-blockers, calcium channel blockers, antiplatelet drugs, statins, angiotensin-converting enzyme inhibitors, angiotensin II receptor antagonists, and, if necessary, revascularization therapy. The patients in the JWZX granules treatment group, in addition to standard treatment, were administered 1 packet of JWZX granules (Jiuwei, approval number Z20080008, Beijing Beilu Pharmaceutical Co., Ltd., Beijing, China) dissolved in warm water, 3 times a day for a duration of 3 months.

### 2.3. Clinical outcome

We also collected the clinical characteristics of the included patients, such as age, gender, body mass index, disease duration, and comorbidities, were collected as demographic and clinical data. Additionally, psychological questionnaire scores, including the HAMA, HAMD, SAS, SDS, GAD-7, and PHQ-9, were collected for all patients before and after treatment. Furthermore, any of the following events occurring during the 1-year follow-up period were defined as major adverse cardiovascular events (MACE) in the poor prognosis group: cardiac death, heart failure, cardiogenic shock, recurrent myocardial infarction, and arrhythmias accompanied by hemodynamic instability.

### 2.4. Enzyme-linked immunosorbent assay (ELISA)

Serum levels of TREM1, brain-derived neurotrophic factor (BDNF), and 5-hydroxytryptamine (5-HT) were measured using commercially available ELISA kits before and after 3 months of treatment. Blood samples (5 mL) were collected from all CHD patients via venipuncture after an overnight fast. The samples were allowed to clot at room temperature for 30 minutes and then centrifuged at 2000 × g for 15 minutes at 4 °C to separate the serum. The supernatant was carefully collected and stored at −80 °C until analysis. For the detection of TREM1, BDNF, and 5-HT, serum samples were thawed on ice, and the assays were performed according to the manufacturer’s instructions. The optical density of each well was measured at 450 nm using a microplate reader, and the concentrations of TREM1, BDNF, and 5-HT were calculated based on standard curves. All samples were tested duplicate to ensure accuracy. The commercially available ELISA kits were: TREM1 (MBS701880, MyBioSource, San Diego), 5-HT (MBS266457, MyBioSource, San Diego), BDNF (MBS8420386, MyBioSource, San Diego).

### 2.5. Statistical analysis

Data analysis was performed using SPSS 25.0 statistical software (IBM, Armonk). The normality of the data was confirmed using the Kolmogorov–Smirnov test. Normally distributed data were expressed as mean ± standard deviation, while non-normally distributed data were expressed as median (range). Between-group comparisons were conducted using the Mann–Whitney test or Student *t* test. Proportions were compared using the Chi-square test. Correlation analysis was performed using Pearson rank correlation or Spearman rank correlation. Receiver operating characteristic (ROC) curves were used to analyze the predictive role of serum TREM1 in the development of MACE in CHD patients. Logistic regression analysis was conducted to determine the risk factors for poor prognosis in CHD patients. Differences with a *P*-value < .05 were considered statistically significant.

## 3. Results

### 3.1. Baseline characteristics of all patients

This study included 50 patients with CHD treated with JWZX pellets (JWZX group) and 50 patients with CHD treated with conventional therapy during the same period (conventional group). As shown in Table 1, the JWZX group had a mean age of 51.68 ± 11.40, with 24.0% being female, and an average disease duration of 6 (1–11) years. The conventional group had a mean age of 48.86 ± 11.86, with 54.0% being female, and an average disease duration of 6 (1–12) years. Additionally, 38.0% of patients in the JWZX group had hypertension, 14.0% had diabetes, while 42.0% of patients in the conventional group had hypertension, and 12.0% had diabetes. There were no significant differences in baseline characteristics between the 2 groups.

**Table 1:** Demographic and clinical data of all subjects.

Variable	JWZX group, n = 50	Conventional group, n = 50	*P*
Age (yr)	51.68 ± 11.40	48.86 ± 11.86	.228
Sex, female (%)	24 (48.0)	27 (54.0)	.480
BMI	24.43 ± 2.09	24.73 ± 1.97	.467
Disease duration (yr)	6 (1–11)	6 (1–12)	.688
Hypertension, n (%)	19 (38.0)	21 (42.0)	.665
Diabetes, n (%)	7 (14.0)	6 (12.0)	.834
HAMA	10 (6–15)	9.5 (5–15)	.415
HAMD	13.90 ± 4.09	13.20 ± 4.41	.413
SAS	44.88 ± 9.87	45.54 ± 10.26	.797
SDS	50.60 ± 10.93	50.36 ± 10.74	.259
GAD-7	8.5 (2–12)	6.5 (2–12)	.229
PHQ-9	9.78 ± 2.67	8.60 ± 3.64	.068
TREM1 (pg/mL)	109.33 ± 10.84	107.26 ± 11.77	.361
BDNF (pg/mL)	63.96 ± 10.25	65.91 ± 9.70	.332
5-HT (ng/mL)	20.25 ± 3.96	19.38 ± 4.53	.309

Continuous data presented non-normal distribution were expressed by median (minimum to maximum) and analyzed by Mann–Whitney *U* test. Continuous data presented normal distribution were expressed by mean ± SD and analyzed by Student *t* test. Chi square test was used for rates.

5-HT = 5-hydroxytryptamine, BDNF = brain-derived neurotrophic factor, BMI = body mass index, GAD-7 = Generalized Anxiety Disorder Scale-7, HAMA = Hamilton Anxiety Scale, HAMD = Hamilton Depression Scale, PHQ-9 = Patient Health Questionnaire-9, SAS = Self-rating Anxiety Scale, SDS = Self-rating Depression Scale, TREM1 = triggering receptor expressed on myeloid cells 1.

### 3.2. JWZX granules treatment improves depression and anxiety

**Figure 1.**
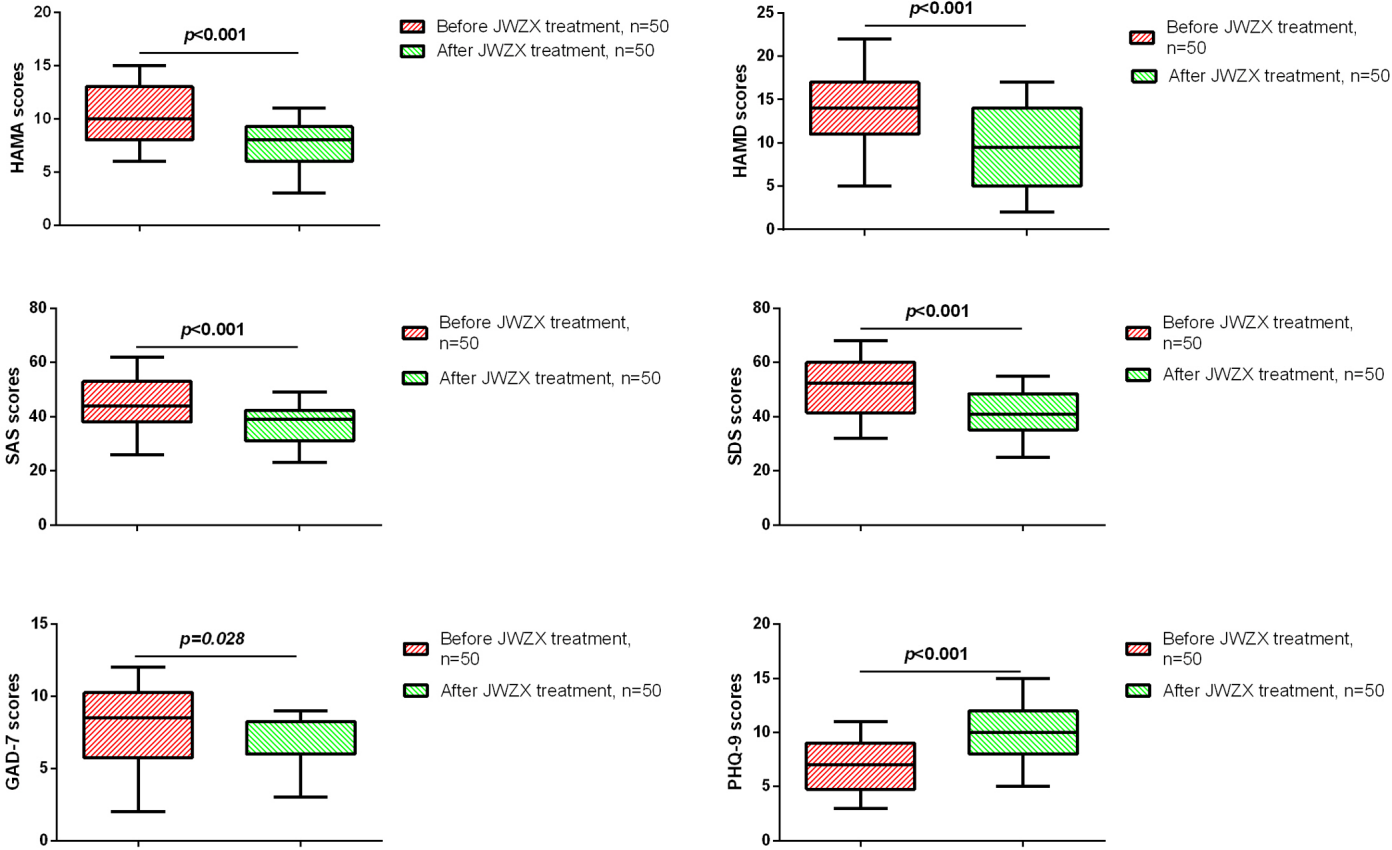
Comparison of scale scores before and after JWZX treatment. JWZX = Jiuwei Zhenxin.

**Figure 2.**
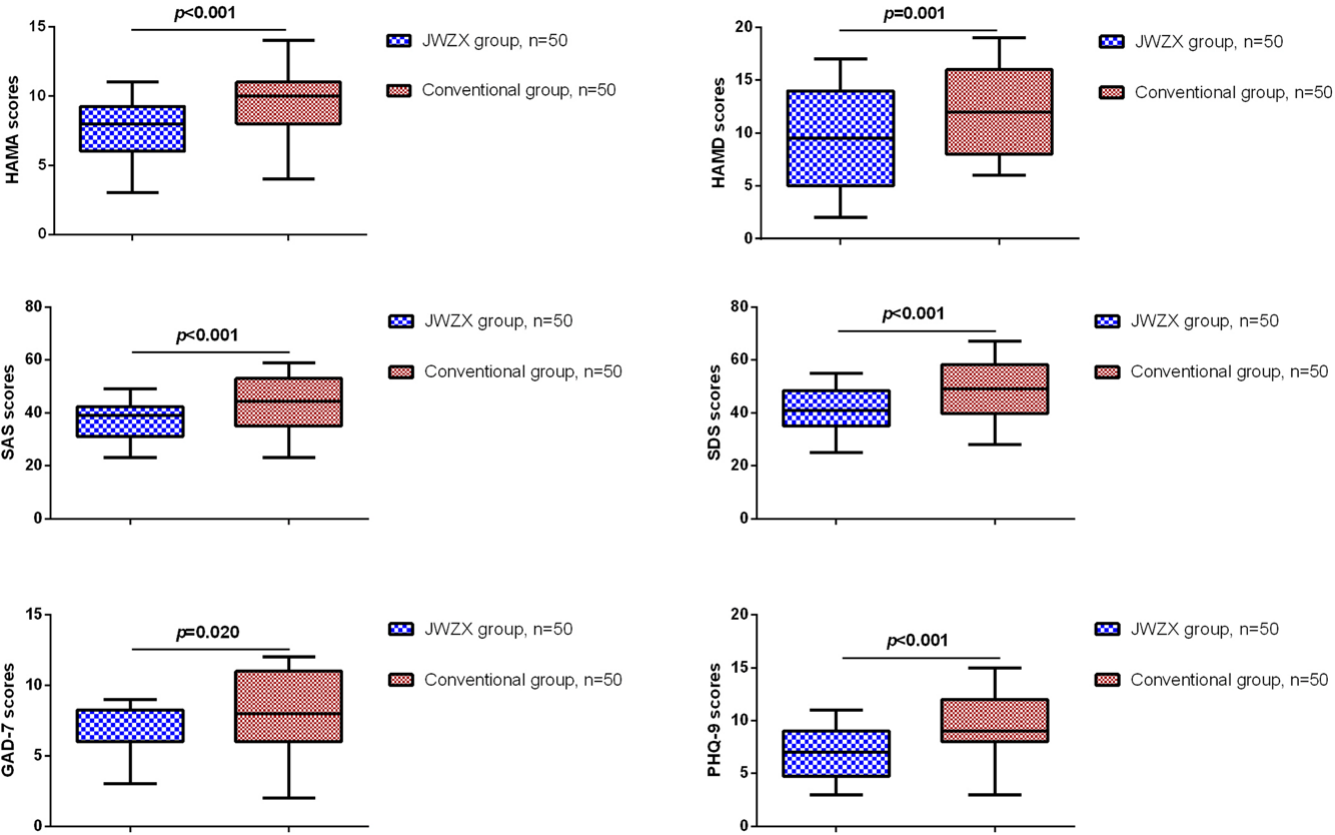
Comparison of scale scores between JWZX treatment and conventional treatment. JWZX = Jiuwei Zhenxin.

Subsequently, we compared the HAMA, HAMD, SAS, SDS, GAD-7, and PHQ-9 questionnaire scores of patients in the JWZX group before and after treatment. As shown in Figure 1, after JWZX treatment, CHD patients showed significant reductions in HAMA, HAMD, SAS, SDS, GAD-7, and PHQ-9 scores. Furthermore, compared to the conventional treatment group, patients in the JWZX group showed significant reductions in HAMA, HAMD, SAS, SDS, GAD-7, and PHQ-9 scores (Fig. 2).

### 3.3. JWZX granules treatment reduces serum TREM1 levels in CHD patients

**Figure 3.**
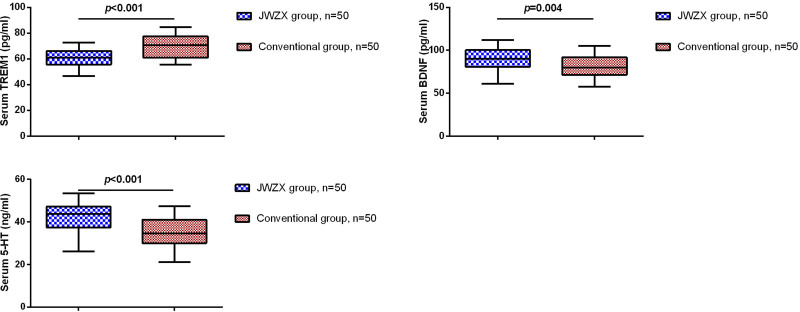
Comparison of serum TREM1, BDNF, and 5-HT levels between JWZX treatment and conventional treatment. 5-HT = 5-hydroxytryptamine, BDNF = brain-derived neurotrophic factor, JWZX = Jiuwei Zhenxin, TREM1 = triggering receptor expressed on myeloid cells 1.

We further compared the serum levels of TREM1, BDNF, and 5-HT in all CHD patients after 3 months of treatment. As shown in Figure 3, compared to the conventional treatment group, patients in the JWZX group exhibited a significant reduction in serum TREM1 levels, while serum BDNF and 5-HT levels were significantly increased. Pearson correlation analysis revealed that serum TREM1 levels were negatively correlated with 5-HT levels, whereas serum BDNF levels were positively correlated with 5-HT levels (Table 2).

**Table 2:** Correlation of serum TREM1, BDNF, and 5-HT levels.

		TREM1	BDNF	5-HT
TREM1	Pearson correlation	1	−0.128	−0.269
	*P*		.203	.007
BDNF	Pearson correlation	−0.128	1	0.236
	*P*	.203		.018
5-HT	Pearson correlation	−0.269	0.236	1
	*P*	.007	.018	

5-HT = 5-hydroxytryptamine, BDNF = brain-derived neurotrophic factor, TREM1 = triggering receptor expressed on myeloid cells 1.

### 3.4. Subgroup analysis

Further subgroup analysis was performed by dividing all patients into high and low HAMA groups and high and low HAMD groups, based on the average HAMA and HAMD scores, respectively. As shown in Table 3, patients in the high HAMA group had significantly higher scores on SAS, SDS, GAD-7, and PHQ-9 compared to those in the low HAMA group. Additionally, compared to patients in the low HAMD group, patients in the high HAMD group had significantly higher SDS, GAD-7 scores and higher serum TREM1 levels (Table 4). In both subgroup analyses, we found that the proportion of CHD patients receiving JWZX treatment was significantly higher in the low HAMD and low HAMA groups.

**Table 3:** Subgroup analysis of HAMA scores.

Variable	Low HAMA scores group, n = 60	High HAMA scores group, n = 40	*P*
JWZX treatment, n (%)	38 (63.3)	12 (30.0)	<.001
HAMD	10.5 (2–19)	10.5 (5–19)	.147
SAS	38.53 ± 8.96	42.68 ± 10.10	.034
SDS	43.02 ± 9.25	47.23 ± 10.58	.039
GAD-7	7 (2–12)	8 (2–12)	.034
PHQ-9	8 (3–15)	8 (3–14)	.143
TREM1 (pg/mL)	64.76 ± 8.75	65.74 ± 10.15	.661
BDNF (pg/mL)	87.49 ± 13.67	82.41 ± 12.46	.062
5-HT (ng/mL)	39.61 ± 8.68	36.79 ± 7.37	.095

Continuous data presented non-normal distribution were expressed by median (minimum to maximum) and analyzed by Mann–Whitney *U* test. Continuous data presented normal distribution were expressed by mean ± SD and analyzed by Student *t* test. Chi square test was used for rates.

5-HT = 5-Hydroxytryptamine, BDNF = brain-derived neurotrophic factor, GAD-7 = Generalized Anxiety Disorder Scale-7, HAMA = Hamilton Anxiety Scale, HAMD = Hamilton Depression Scale, JWZX = Jiuwei Zhenxin, PHQ-9 = Patient Health Questionnaire-9, SAS = Self-rating Anxiety Scale, SDS = Self-rating Depression Scale, TREM1 = Triggering Receptor Expressed on Myeloid Cells 1.

**Table 4:** Subgroup analysis of HAMD scores.

Variable	Low HAMD scores group, n = 56	High HAMD scores group, n = 44	*P*
JWZX treatment, n (%)	32 (57.1)	18 (40.9)	.034
HAMA	8 (3–14)	9 (4–13)	.569
SAS	39.34 ± 9.13	41.27 ± 10.17	.320
SDS	43.04 ± 9.72	47.27 ± 10.01	.035
GAD-7	7 (2–12)	7.5 (2–12)	.033
PHQ-9	8.11 ± 2.67	8.39 ± 3.42	.143
TREM1 (pg/mL)	62.64 ± 9.68	67.82 ± 8.85	.029
BDNF (pg/mL)	87.69 ± 13.10	82.62 ± 13.31	.059
5-HT (ng/mL)	38.08 ± 8.06	39.00 ± 8.58	.588

Continuous data presented non-normal distribution were expressed by median (minimum to maximum) and analyzed by Mann–Whitney *U* test. Continuous data presented normal distribution were expressed by mean ± SD and analyzed by Student *t* test. Chi square test was used for rates.

5-HT = 5-Hydroxytryptamine, BDNF = brain-derived neurotrophic factor, GAD-7 = Generalized Anxiety Disorder Scale-7, HAMA = Hamilton Anxiety Scale, HAMD = Hamilton Depression Scale, JWZX = Jiuwei Zhenxin, PHQ-9 = Patient Health Questionnaire-9, SAS = Self-rating Anxiety Scale, SDS = Self-rating Depression Scale, TREM1 = Triggering Receptor Expressed on Myeloid Cells 1.

### 3.5. ROC curve analysis of serum TREM1 for predicting MACE in CHD patients

**Figure 4.**
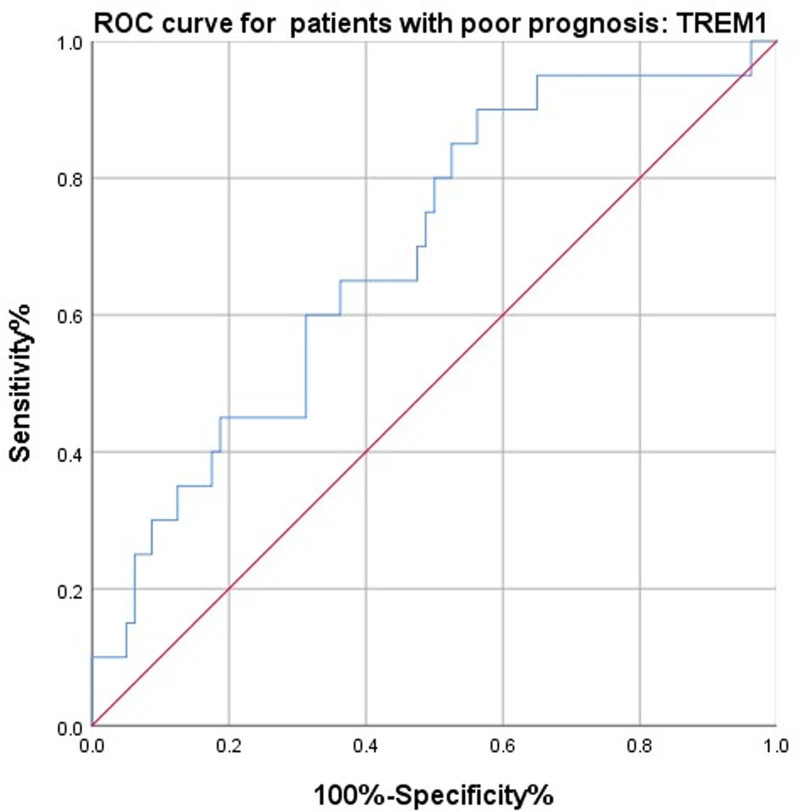
ROC curve analysis of serum TREM1 for predicting MACE in CHD patients. CHD = coronary heart disease, MACE = major adverse cardiovascular events, ROC = receiver operating characteristic, TREM1 = triggering receptor expressed on myeloid cells 1.

The predictive value of serum TREM1 for MACE within 1 year after treatment in CHD patients was evaluated using ROC curve analysis. As shown in Figure 4, posttreatment serum TREM1 demonstrated a moderate predictive value for MACE occurrence within 1 year. The area under the curve for serum TREM1 in predicting MACE was 0.689, with a cutoff value of 66.39 pg/mL, a sensitivity of 65.0%, and a specificity of 63.7%.

### 3.6. Logistic regression analysis for adverse prognosis in CHD patients

To explore the risk factors for adverse prognosis in all CHD patients, patients who experienced MACE during a 1-year follow-up were defined as the adverse prognosis group. Logistic regression analysis was used to identify the factors associated with adverse prognosis in patients at 1-year. As shown in Table 5, logistic regression analysis revealed that disease duration, JWZX treatment, HAMD before treatment and serum TREM1, 5-HT levels after treatment were significant factors associated with patient prognosis.

**Table 5:** Logistic regression for risk factors of adverse prognosis in CHD patients.

Variables	Wald	Odds ratio	95% CI	*P*
Model 1				
JWZX treatment	5.670	4.147	1.286–13.373	.017
Age	0.039	1.005	0.956–1.057	.844
Sex	0.025	0.913	0.298–1.801	.874
BMI	1.923	1.224	0.980–1.629	.166
Disease duration	3.981	1.223	1.004–1.490	.046
Hypertension	1.623	0.450	0.132–1.537	.203
Diabetes	0.865	2.055	0.450–9.374	.352
Model 2				
HAMA before treatment	1.835	0.838	0.649–1.082	.176
HAMD before treatment	4.423	1.236	1.015–1.506	.035
SAS before treatment	0.415	0.979	0.919–1.043	.520
SDS before treatment	0.519	0.978	0.920–1.039	.471
GAD-7 before treatment	0.144	1.047	0.827–1.324	.704
PHQ-9 before treatment	0.162	0.957	0.774–1.184	.687
HAMA after treatment	0.435	1.098	0.832–1.448	.510
HAMD after treatment	0.009	0.992	0.842–1.169	.925
SAS after treatment	1.634	0.948	0.874–1.029	.201
SDS after treatment	1.796	1.049	0.978–1.124	.180
GAD-7 after treatment	2.455	1.278	0.940–1.737	.117
PHQ-9 after treatment	0.882	1.110	0.893–1.379	.348
TREM1 before treatment	0.849	0.973	0.919–1.031	.357
BDNF before treatment	0.082	0.990	0.926–1.059	.775
5-HT before treatment	0.481	0.947	0.811–1.105	.488
TREM1 after treatment	4.567	1.081	1.006–1.161	.033
BDNF after treatment	0.003	1.001	0.949–1.056	.958
5-HT after treatment	5.406	0.899	0.822–0.983	.020

5-HT = 5-Hydroxytryptamine, BDNF = brain-derived neurotrophic factor, BMI = body mass index, GAD-7 = Generalized Anxiety Disorder Scale-7, HAMA = Hamilton Anxiety Scale, HAMD = Hamilton Depression Scale, JWZX = Jiuwei Zhenxin, PHQ-9 = Patient Health Questionnaire-9, SAS = Self-rating Anxiety Scale, SDS = Self-rating Depression Scale, TREM1 = Triggering Receptor Expressed on Myeloid Cells 1.

## 4. Discussion

In recent years, depression has become an increasingly serious global problem and has been consistently associated with an increased risk of CHD.^[18]^ The prevalence of depression in patients with coronary heart disease is 2 to 3 times higher than in the general population, with rates ranging from 15% to 30%.^[19]^ This highlights the importance of addressing psychological issues in CHD patients, as they may significantly impact both treatment outcomes and prognosis. In this study, we investigated the effects of JWZX granules on serum TREM1 levels and their regulatory role in depression and anxiety in CHD patients. Our findings demonstrated that JWZX treatment not only improved psychological symptoms but also significantly reduced serum TREM1 levels, which were negatively correlated with 5-HT levels. These results suggested that JWZX granules might modulate neuroinflammation and neurotransmitter activity, offering a potential therapeutic approach for improving psychological health in CHD patients.

Previous research has already focused on the role of psychological factors in CHD patients. Psychological factors such as depression and anxiety may lead to unhealthy lifestyle behaviors in patients, such as poor diet and lack of exercise, thereby increasing the risk of developing the disease.^[20]^ There are also reports showing that depression and anxiety are associated with increased risk of CHD incidence, recurrence, and mortality.^[21,22]^ Additionally, depression and anxiety often result in decreased quality of life for CHD patients, with feelings of low mood, lack of motivation and pleasure, as well as impairments in social and family functioning.^[23]^ We also found in our study that baseline HAMD was a risk factor for adverse prognosis in CHD patients, indicating that improving depression and anxiety in CHD patients may have positive effects on their treatment and prognosis. Previous research has been dedicated to improving the psychological well-being of CHD patients. For example, a meta-analysis by Albus et al included 20 studies involving a total of 4450 coronary heart disease patients (88.5%) or congestive heart failure patients (11.5%), and the analysis showed that specific psychological interventions provided during exercise-based cardiac rehabilitation may help reduce depressive symptoms and cardiac morbidity.^[24]^ Another study demonstrated that depression/anxiety was associated with increased risk of mortality in adult congenital heart disease patients, and addressing the social and psychological needs of patients may have a significant impact on optimizing healthcare service utilization and improving prognosis.^[25]^ The latest randomized controlled trial also confirmed that the addition of inulin to probiotic supplementation was more effective in improving psychological factors and inflammatory biomarkers in patients with coronary artery disease (CAD) compared to the supplementation of each component alone.^[26]^

JWZX is a traditional Chinese herbal medicine,^[12]^ which contains various active ingredients such as ginsenosides, Rehmannia-related polysaccharides, jujuboside, and paeonol glucoside, which have been shown in animal experiments to have antidepressant, anxiolytic, and neuroprotective properties.^[27,28]^ In clinical studies, a randomized controlled trial by Wang et al confirmed that JWZX is an effective, safe, and well-tolerated treatment for anxiety.^[13]^ Another study demonstrated that JWZX granules combined with fluvoxamine treatment significantly improved the symptoms of geriatric depression, alleviated sleep disturbances, reduced suicide risk, relieved caregiver burden, and improved the quality of life, showing promising clinical application.^[13]^ The results of a study by Xia et al showed that JWZX combined with buspironone hydrochloride tablets reduced anxiety symptoms in elderly patients with anxiety disorder, and the mechanism may be related to the modulation of neurotransmitters and inhibition of autonomic nervous system dysfunction.^[29]^ However, there have been limited studies focusing on the therapeutic effects of JWZX on anxiety and depression in patients. To our knowledge, we are the first to discover that JWZX treatment significantly reduced depression and anxiety in CHD patients and improved patient prognosis.

Additionally, our study provides novel insights into the role of serum TREM1 as a critical biomarker linking neuroinflammation, psychological symptoms, and cardiovascular prognosis in CHD patients. The observed negative correlation between serum TREM1 and 5-HT levels aligned with emerging evidence that neuroinflammatory processes, mediated by receptors like TREM1, might disrupt neurotransmitter homeostasis.^[30]^ Specifically, elevated TREM1 levels in patients with high HAMD scores suggested its involvement in depressive pathophysiology, potentially through amplifying neuroinflammatory cascades. Preclinical studies have demonstrated that TREM1 inhibition using LP17 alleviated depressive-like behaviors by attenuating neuroinflammation in the prefrontal cortex and restoring neuronal activity through the PI3K/Akt pathway.^[16]^ This aligns with our findings where elevated TREM1 levels in high-HAMD patients correlated with worsened depressive symptoms, suggesting TREM1-driven neuroinflammation as a critical mediator of serotonin dysregulation. Furthermore, Wu et al revealed that TREM1 overexpression in a colitis model exacerbated visceral hypersensitivity and depressive-like behaviors by amplifying microglial activation in the anterior cingulate cortex, while TREM1 deficiency mitigated these effects.^[31]^ This underscored TREM1’s dual role in both peripheral and central inflammatory cascades, which might disrupt 5-HT synthesis via pro-inflammatory cytokines that inhibited tryptophan hydroxylase activity. Together, these studies highlighted TREM1 as a pivotal regulator of neuroimmune crosstalk, linking systemic inflammation to serotonin metabolism and depressive pathophysiology.

Moreover, the predictive value of serum TREM1 for MACE within 1 year further highlights its dual role in both psychological and cardiovascular pathology. Elevated TREM1 levels may reflect a persistent pro-inflammatory state that destabilizes atherosclerotic plaques or promotes endothelial dysfunction, thereby increasing cardiovascular risk.^[32]^ Our ROC analysis (area under the curve: 0.689) and logistic regression results, which identified TREM1 as an independent prognostic factor, corroborate findings from cardiovascular studies implicating TREM1 in adverse outcomes through its amplification of inflammatory responses.^[33]^ Notably, the cutoff value of 66.39 pg/mL offers a potential threshold for clinical risk stratification, though further validation is warranted. These results collectively position TREM1 as a bridge between neuropsychiatric and cardiovascular morbidity in CHD patients, emphasizing the need for therapies targeting inflammatory pathways to address both domains. Future studies should explore whether direct TREM1 inhibition, combined with interventions like JWZX granules, could synergistically improve psychological and cardiovascular outcomes in this high-risk population.

However, this study has several limitations. Firstly, it is a single-center study, which may limit the generalizability of the findings. Secondly, the sample size was relatively small, potentially reducing the statistical power to detect subtle effects. Thirdly, while we observed significant improvements in psychological factors and serum biomarkers (e.g., TREM1, 5-HT, and BDNF) in CHD patients, the study did not include animal experiments to validate the molecular mechanisms underlying these effects. Future research should incorporate animal models to further elucidate the pathways through which JWZX granules modulate neuroinflammation and neurotransmitter activity, particularly focusing on TREM1 and its downstream signaling pathways. Additionally, longitudinal studies with larger cohorts are needed to confirm the long-term benefits of JWZX treatment on both psychological and cardiovascular outcomes in CHD patients.

## 5. Conclusion

Our research demonstrated that JWZX treatment significantly reduced depression and anxiety in CHD patients and modulates serum TREM1 levels. Additionally, serum TREM1 showed predictive value for MACE, highlighting its potential as a biomarker for cardiovascular risk stratification. These findings supported the use of JWZX granules as a complementary therapy for CHD patients with psychological comorbidities. Future studies should further explore the molecular mechanisms of TREM1 modulation to optimize therapeutic strategies.

## Author contributions

**Conceptualization:** Chun Guo, Yi Xiang.

**Data curation:** Yi Xiang, Shan Hui, Zhongguang Yan.

**Formal analysis:** Yi Xiang, Shan Hui.

**Methodology:** Shan Hui.

**Project administration:** Chun Guo.

**Software:** Hao Nie.

**Supervision:** Chun Guo.

**Writing – review & editing:** Chun Guo.

**Writing – original draft:** Yi Xiang.

## References

[1] Arnett DK Blumenthal RS Albert MA. 2019 ACC/AHA Guideline on the Primary Prevention of Cardiovascular Disease: a report of the American College of Cardiology/American Heart Association Task Force on Clinical Practice Guidelines. Circulation. 2019;140:e596–646. 30879355 10.1161/CIR.0000000000000678 PMC7734661

[2] Mazloumi E Poorolajal J Sarrafzadegan N Roohafza HR Faradmal J Karami M. Avoidable burden of cardiovascular diseases in the eastern mediterranean region: contribution of selected risk factors for cardiovascular-related deaths. High Blood Press Cardiovasc Prev. 2019;26:227–37. 31228169 10.1007/s40292-019-00319-3

[3] Brown A Kritharides L. Cardiovascular disease. Heart Lung Circ. 2010;19:263. 20363188 10.1016/j.hlc.2010.02.012

[4] Magnussen C Ojeda FM Leong DP. Global effect of modifiable risk factors on cardiovascular disease and mortality. N Engl J Med. 2023;389:1273–85. 37632466 10.1056/NEJMoa2206916 PMC10589462

[5] Teo KK Rafiq T. Cardiovascular risk factors and prevention: a perspective from developing countries. Can J Cardiol. 2021;37:733–43. 33610690 10.1016/j.cjca.2021.02.009

[6] Worthley SG Osende JI Helft G Badimon JJ Fuster V. Coronary artery disease: pathogenesis and acute coronary syndromes. Mt Sinai J Med. 2001;68:167–81. 11373689

[7] Ma LY Chen WW Gao RL. China cardiovascular diseases report 2018: an updated summary. J Geriatr Cardiol. 2020;17:1–8. 32133031 10.11909/j.issn.1671-5411.2020.01.001 PMC7008101

[8] Ren Y Yang H Browning C Thomas S Liu M. Prevalence of depression in coronary heart disease in China: a systematic review and meta-analysis. Chin Med J (Engl). 2014;127:2991–8. 25131240

[9] Cai X Zhou J Li W Cheng L Yuan Z Xiao Y. Potential influential factors of in-hospital myocardial reinfarction in ST-segment elevation myocardial infarction (STEMI) patients: finding from the improving care for cardiovascular disease in China- (CCC-) acute coronary syndrome (ACS) Project. Oxid Med Cell Longev. 2021;2021:9977312. 34659644 10.1155/2021/9977312 PMC8514929

[10] Cheng M Lei X Zhu C. The association between poor sleep quality and anxiety and depression symptoms in Chinese patients with coronary heart disease. Psychol Health Med. 2022;27:1347–56. 33506709 10.1080/13548506.2021.1874440

[11] Peter RS Meyer ML Mons U. Long-term trajectories of anxiety and depression in patients with stable coronary heart disease and risk of subsequent cardiovascular events. Depress Anxiety. 2020;37:784–92. 32237189 10.1002/da.23011

[12] Wang J Wu X. Traditional Chinese medicine Jiuwei Zhenxin granules in treating depression: an overview. Neuropsychiatr Dis Treat. 2020;16:2237–55. 33116523 10.2147/NDT.S273324 PMC7541918

[13] Wang X Chen P Shi S. Effectiveness and safety of Jiuwei Zhenxin granules for treating generalized anxiety disorder: a randomized controlled trial. Front Psychiatry. 2022;13:898683. 36267853 10.3389/fpsyt.2022.898683 PMC9576854

[14] Kouassi KT Gunasekar P Agrawal DK Jadhav GP. TREM-1; is it a pivotal target for cardiovascular diseases. J Cardiovasc Dev Dis. 2018;5:45. 30205488 10.3390/jcdd5030045 PMC6162371

[15] Xu P Hong Y Xie Y. TREM-1 exacerbates neuroinflammatory injury via NLRP3 inflammasome-mediated pyroptosis in experimental subarachnoid hemorrhage. Transl Stroke Res. 2021;12:643–59. 32862402 10.1007/s12975-020-00840-x

[16] Fu A Qiao F Feng H Luo Q. Inhibition of TREM-1 ameliorates lipopolysaccharide-induced depressive-like behaviors by alleviating neuroinflammation in the PFC via PI3K/Akt signaling pathway. Behav Brain Res. 2023;449:114464. 37142164 10.1016/j.bbr.2023.114464

[17] Montalescot G Sechtem U Achenbach S. 2013 ESC guidelines on the management of stable coronary artery disease: the Task Force on the management of stable coronary artery disease of the European Society of Cardiology. Eur Heart J. 2013;34:2949–3003. 23996286 10.1093/eurheartj/eht296

[18] Whiteford HA Degenhardt L Rehm J. Global burden of disease attributable to mental and substance use disorders: findings from the Global Burden of Disease Study 2010. Lancet. 2013;382:1575–86. 23993280 10.1016/S0140-6736(13)61611-6

[19] Vaccarino V Badimon L Bremner JD. Depression and coronary heart disease: 2018 position paper of the ESC working group on coronary pathophysiology and microcirculation. Eur Heart J. 2020;41:1687–96. 30698764 10.1093/eurheartj/ehy913 PMC10941327

[20] Bonnet F Irving K Terra JL Nony P Berthezène F Moulin P. Anxiety and depression are associated with unhealthy lifestyle in patients at risk of cardiovascular disease. Atherosclerosis. 2005;178:339–44. 15694943 10.1016/j.atherosclerosis.2004.08.035

[21] Janszky I Ahnve S Lundberg I Hemmingsson T. Early-onset depression, anxiety, and risk of subsequent coronary heart disease: 37-year follow-up of 49,321 young Swedish men. J Am Coll Cardiol. 2010;56:31–7. 20620714 10.1016/j.jacc.2010.03.033

[22] Watkins LL Koch GG Sherwood A. Association of anxiety and depression with all-cause mortality in individuals with coronary heart disease. J Am Heart Assoc. 2013;2:e000068. 23537805 10.1161/JAHA.112.000068 PMC3647264

[23] Foxwell R Morley C Frizelle D. Illness perceptions, mood and quality of life: a systematic review of coronary heart disease patients. J Psychosom Res. 2013;75:211–22. 23972409 10.1016/j.jpsychores.2013.05.003

[24] Albus C Herrmann-Lingen C Jensen K. Additional effects of psychological interventions on subjective and objective outcomes compared with exercise-based cardiac rehabilitation alone in patients with cardiovascular disease: a systematic review and meta-analysis. Eur J Prev Cardiol. 2019;26:1035–49. 30857429 10.1177/2047487319832393 PMC6604240

[25] Benderly M Kalter-Leibovici O Weitzman D. Depression and anxiety are associated with high health care utilization and mortality among adults with congenital heart disease. Int J Cardiol. 2019;276:81–6. 30224258 10.1016/j.ijcard.2018.09.005

[26] Moludi J Khedmatgozar H Nachvak SM. The effects of co-administration of probiotics and prebiotics on chronic inflammation, and depression symptoms in patients with coronary artery diseases: a randomized clinical trial. Nutr Neurosci. 2022;25:1659–68. 33641656 10.1080/1028415X.2021.1889451

[27] Liu D Zhang H Gu W Liu Y Zhang M. Neuroprotective effects of ginsenoside Rb1 on high glucose-induced neurotoxicity in primary cultured rat hippocampal neurons. PLoS One. 2013;8:e79399. 24223941 10.1371/journal.pone.0079399 PMC3815219

[28] Zheng M Xin Y Li Y. A potential neuroprotective agent. Biomed Res Int. 2018;2018:8174345. 29854792 10.1155/2018/8174345 PMC5964429

[29] Xia M Liu X Zhu H Ji X. Research on the clinical effect of Jiuwei-Zhenxin granules and buspirone hydrochloride tablets for geriatric anxiety patients. Int J Tradit Chin Med. 2019;6:1170–3.

[30] Li H Yu W Zheng X Zhu Z. TREM1-microglia crosstalk: neurocognitive disorders. Brain Res Bull. 2025;220:111162. 39645047 10.1016/j.brainresbull.2024.111162

[31] Wu K Liu YY Shao S. The microglial innate immune receptors TREM-1 and TREM-2 in the anterior cingulate cortex (ACC) drive visceral hypersensitivity and depressive-like behaviors following DSS-induced colitis. Brain Behav Immun. 2023;112:96–117. 37286175 10.1016/j.bbi.2023.06.003

[32] Chen X Yu L Meng S. Inhibition of TREM-1 ameliorates angiotensin II-induced atrial fibrillation by attenuating macrophage infiltration and inflammation through the PI3K/AKT/FoxO3a signaling pathway. Cell Signal. 2024;124:111458. 39384003 10.1016/j.cellsig.2024.111458

[33] Wang YK Tang JN Shen YL. Prognostic utility of soluble TREM-1 in predicting mortality and cardiovascular events in patients with acute myocardial infarction. J Am Heart Assoc. 2018;7:e008985. 29886421 10.1161/JAHA.118.008985 PMC6220534

